# Toxicology knowledge graph for structural birth defects

**DOI:** 10.1038/s43856-023-00329-2

**Published:** 2023-07-17

**Authors:** John Erol Evangelista, Daniel J. B. Clarke, Zhuorui Xie, Giacomo B. Marino, Vivian Utti, Sherry L. Jenkins, Taha Mohseni Ahooyi, Cristian G. Bologa, Jeremy J. Yang, Jessica L. Binder, Praveen Kumar, Christophe G. Lambert, Jeffrey S. Grethe, Eric Wenger, Deanne Taylor, Tudor I. Oprea, Bernard de Bono, Avi Ma’ayan

**Affiliations:** 1 Department of Pharmacological Sciences, Mount Sinai Center for Bioinformatics, Icahn School of Medicine at Mount Sinai, New York, NY 10029 USA; 2 The Children’s Hospital of Philadelphia, Department of Biomedical and Health Informatics; Department of Pediatrics, University of Pennsylvania Perelman School of Medicine, Philadelphia, PA 19104 USA; 3 Department of Internal Medicine, Division of Translational Informatics, University of New Mexico, Albuquerque, NM 87131 USA; 4 Department of Medicine, University of California San Diego, La Jolla, CA 92093 USA; 5 Auckland Bioengineering Institute, University of Auckland, Auckland, New Zealand

**Keywords:** Databases, Embryogenesis, Reproductive disorders, Drug development, Disease genetics

## Abstract

**Background:**

Birth defects are functional and structural abnormalities that impact about 1 in 33 births in the United States. They have been attributed to genetic and other factors such as drugs, cosmetics, food, and environmental pollutants during pregnancy, but for most birth defects there are no known causes.

**Methods:**

To further characterize associations between small molecule compounds and their potential to induce specific birth abnormalities, we gathered knowledge from multiple sources to construct a reproductive toxicity Knowledge Graph (ReproTox-KG) with a focus on associations between birth defects, drugs, and genes. Specifically, we gathered data from drug/birth-defect associations from co-mentions in published abstracts, gene/birth-defect associations from genetic studies, drug- and preclinical-compound-induced gene expression changes in cell lines, known drug targets, genetic burden scores for human genes, and placental crossing scores for small molecules.

**Results:**

Using ReproTox-KG and semi-supervised learning (SSL), we scored >30,000 preclinical small molecules for their potential to cross the placenta and induce birth defects, and identified >500 birth-defect/gene/drug cliques that can be used to explain molecular mechanisms for drug-induced birth defects. The ReproTox-KG can be accessed via a web-based user interface available at https://maayanlab.cloud/reprotox-kg. This site enables users to explore the associations between birth defects, approved and preclinical drugs, and all human genes.

**Conclusions:**

ReproTox-KG provides a resource for exploring knowledge about the molecular mechanisms of birth defects with the potential of predicting the likelihood of genes and preclinical small molecules to induce birth defects.

## Plain language summary

While birth defects are common, for most birth defects there are no known causes. During pregnancy, developing babies are exposed to drugs, cosmetics, food, and environmental pollutants that may cause birth defects. However, exactly how these environmental factors are involved in producing birth defects is difficult to discern. Also, birth defects can be a consequence of the genes inherited from the parents. We combined general data about human genes and drugs with specific data previously implicating genes and drugs in inducing birth defects to create a knowledge graph representation that connects genes, drugs, and birth defects. This knowledge graph can be used to explore new links that may explain why birth defects occur, particularly those that result from a combination of inherited and environmental influences.

## Abstract (web summary)

Clarke, Evangelista et al. use a knowledge graph (ReproTox-KG) to characterize associations between small molecule compounds and their potential to induce specific birth abnormalities. They identify over 500 birth defect/gene/drug connections that can explain molecular mechanisms for drug-induced birth defects.

## Introduction

The United States Department of Labor’s Occupational Safety and Health Administration^1^ defines reproductive toxicity as a characteristic of substances or agents that may affect the reproductive health of women or men or the ability of couples to have healthy children. These hazards may cause problems such as infertility, miscarriage, and birth defects. The prevention and clinical management of reproductive toxicity caused by chemical agents^2^ requires the combined expertise from several medical fields, including public health and occupational health to protect against environmental/occupational toxins that lead to miscarriage^3^, food and drug regulatory medicine to avoid drug teratogenicity or toxins in food that impact fertility, as well as clinical genetics, obstetrics, gynecology, and pediatrics to screen, prevent, monitor, and manage birth defects. This multidisciplinary nature of reproductive health is challenging. For instance, prescribing drugs in pregnancy remains a complex and controversial issue for both pregnant persons and physicians^4^. Prescriptions given to to pregnant persons of drugs known to potentially cause birth defects based on evidence from animal models, human studies, and based on post-marketing evidence is a frequent event. A cohort study of over a million pregnant persons found that 42% had at least one prescription for such medications^5^. A key challenge to prescribing for the gravid patient is that recommendations are based on limited human pharmacological data and conflicting cases of adverse outcomes, given that pregnant persons are routinely excluded from randomized controlled trials^6^. Combinatorial complexity challenges and data availability limitations are also key considerations in the prediction of drug–drug interactions^7,8^ that may impact reproductive health^9,10^. It is likely that some birth defects may be caused by a combination of factors that may include interactions between genetics, drugs, and viral or bacterial infections, making it difficult to identify a specific cause. As a result, the causes of birth defects, which in the US account for an estimated 3% of births^1^ and 20% of infant deaths^11,12^, are still mostly poorly understood.

In recent years, knowledge graphs have gained popularity as a productive approach to integrate data from multiple sources to organize information and glean new knowledge^13^. Knowledge graph databases store information about the semantic relationships between objects and represent events as triples: subject->predicate->object, for example, chicken->lays->eggs. Once these assertions are combined, they form a network made of nodes and edges and this establishes the knowledge graph. Once data from multiple sources are organized in a knowledge graph, it can be queried to extract subgraphs that can illuminate unexpected associations between entities. Integrated data organized into knowledge graphs can also be used as input into graph embedding algorithms^14,15^ that aim to predict missing associations not present in the original knowledge graph. Such an approach is increasingly applied in the domain of drug discovery^16^. For example, there are efforts that combine drug reaction knowledge encoded in a knowledge graph with side effects information from databases such as the FDA Adverse Event Reporting System (FAERS) to predict adverse events^17,18^ or encoding pharmacogenomics data that connect drugs, variants, and adverse events to explain variant-adverse event associations^19^. In many cases, these types of efforts are derivatives of comprehensive projects that aim to abstract biomedical knowledge-bases into triples or gene-attribute associations, and make such integrated data available for search and knowledge imputation^20^. There are currently several options for knowledge graph databases, including Neo4J^21^, Stardog^22^, ArangoDB^23^, Amazon Neptune^24^, and OrientDB^25^. In this study, we endeavored to combine knowledge about birth defects with knowledge about genes and drugs to identify potential molecular mechanisms for known birth defects and predict birth defects for preclinical drugs and other small molecules. We ranked genes based on their association with pathogenicity; predicted the likelihood of small molecules to cross the placental barrier and induce birth defects using semi-supervised learning; assembled knowledge about known drug targets for marked drugs;^26^ and abstracted knowledge about the effects of drugs and preclinical small molecules on gene expression^27^. All these data are serialized into a knowledge graph representation, stored in a Neo4j database, and provided for access via an original user-friendly web-based user interface. By combining general information about genes, drugs, and preclinical small molecules with knowledge about the association of genes and drugs with birth defects, we were able to predict the likelihood that preclinical compounds will induce birth defects, and whether these compounds are likely to cross the placental barrier. In addition, by analyzing the knowledge graph topological structure, we were able to pinpoint previously unknown associations between drugs and genes based on the birth defects these drugs and genes are known to induce.

## Methods

### Curating phenotypic terms relevant to birth defects

The manual curation of birth defects terms started from a list of observed birth-defect cases from the Gabriella Miller Kids First Pediatric Research Program (Kids First) cohort. This list of real birth defects and their frequencies is provided as supporting materials (Supplementary Data 1) and is available for download from the ReproTox-KG website at the following URL: https://s3.amazonaws.com/maayan-kg/reprotox/HPO_Freq.tsv. The list of observed birth defects had to be pruned to exclude phenotypes that are not specific to birth defects, for example, glioma. Specifically, we focused on abnormal morphologies of the great vessels, heart, and central nervous system (CNS) phenotypes. Using the EMBL-EBI Ontology Lookup Service (OLS) human phenotype ontology (HPO) v2021-10-10 ^28^ we mapped terms from the table of observed cases to HPO identifiers. To this end we considered the parent terms HP:0030962 (Abnormal morphology of the great vessels), HP:0001627 (Abnormal heart morphology) and HP:0012639 (Abnormal nervous system morphology) and extracted all the child nodes. In all, 166, 193, and 177 phenotype terms were retained for great vessels, heart, and CNS, respectively (Supplemental Data 2). The phenotype terms identified as relevant to the heart, large vessels, and CNS were filtered for terms that were not immediately relevant for structural birth defects. Specifically, clinical experts filtered for including phenotypes that could only have developed in utero. So, for example, a term under Conotruncal defect (HP:0001710) would qualify for our list, but not terms related to Cardiomyopathy (HP:0001638), Palpitations (HP:0001962) or Congestive Heart Failure (HP:0001635), even though the latter three phenotypes might also be a secondary consequence of a structural birth defect. In addition, 36 major birth-defect terms were separately extracted from the Centers for Disease Control and Prevention (CDC) website^29^ on January 6, 2022, and manually mapped to HPO identifiers (Supplemental Data 3).

To enhance the consistent representation of the above phenotypic terms, and to link these birth defects with knowledge about the appropriate anatomical entities involved with these pathologies, we manually curated the HPO terms onto an anatomy connectivity knowledge graph. The schema adopted by this graph is based on the ApiNATOMY knowledge representation^30,31^, which was developed as part of the Stimulating Peripheral Activity to Relieve Conditions (SPARC)^32^ connectivity mapping effort. The ApiNATOMY subgraph within the ReproTox-KG provides links to knowledge about constituent anatomical structures such as cell types that may be involved in the birth-defect mechanisms, as well as representations of abnormal anatomical organizations that typify these pathological phenotypes.

### Curating small molecules associated with birth defects

Manually curated teratogens and xenobiotics with potential to cause birth defects were extracted from various sources. We relied on existing resources such as those listed by DrugCentral^33^ and Drugs.com^34^ as FDA D and X category drugs, a report by the National Birth Defects Prevention Study (NBDPS)^35^, a list provided by the National Birth Defects Registry^35^, as well as drugs listed in several other publications^36–40^ (Supplementary Fig. S1 and Supplemental Data 4). In addition, we used DrugShot^41^ which is an automated way to obtain a ranked list of drugs for any search term using PubMed. We also extracted birth-defect/drug associations from the FAERS database via over-representation analysis (Supplemental Data 5). DrugCentral^33^, an online drug information resource, was queried for FDA D and X category drugs and their associated Simplified Molecular Input Line Entry System (SMILES) with absorption, distribution, metabolism, excretion, and toxicity (ADMET) properties. FDA-approved drugs classified as X or D are drugs with evidence of inducing birth defects in humans and animal models. D category drugs are those that,  despite potential risks shown in human studies and postmarketing, may be used in pregnant persons as the potential benefits outweigh the risks, while category X drugs are those that should not be used in pregnant persons because studies in animals or humans have shown fetal abnormalities, and these risks outweigh any potential benefits^42^. In addition, using DrugShot^41^, we queried each CDC birth-defect term through PubMed to extract Pubmed Identifiers (PMIDs) associated with each birth-defect term. Abstracts associated with these PMIDs were mined to extract drug PubChem IDs based on co-mentions of the birth defect with a drug. The DrugShot^41^ method first queries PubMed with drug names to collect PMIDs for each drug. Then we query PubMed with birth-defect terms and counted the overlapping PMIDs between the queries. To normalize for research focus biases, we also include the number of PMIDs returned for the drug and birth defect that do not overlap. The 30 most frequently co-occurring drugs for each birth defect were retained as the drug sets for each birth defect. The cutoff of 30 drugs was set to reasonably ensure that each drug that we retained for the birth defect has multiple publication co-mentions that serve as evidence for such associations. Only the CDC birth defects were used in this part of the analysis. Finally, the list of teratogens from FAERS birth-defect terms were mapped to drugs with a likelihood ratio (LLR) cutoff of LLR > 2 * (likelihood ratio test) LLRT. The formula assumes an over-representation of the presence of the birth-defect term and the drug without considering other factors that may induce the birth defect.

### Evidence implicating genes with birth defects

Given the curated phenotype lists described above, human phenotype–gene associations were retrieved from multiple sources, including Pharos^43^, Online Mendelian Inheritance in Man (OMIM)^44^, Orphanet^45^, ClinVar^46^, DISEASES^47^, DatabasE of genomiC varIation and Phenotype in Humans using Ensembl Resources (DECIPHER)^48^, the American Heart Association (AHA)^49^, and Geneshot^50^. From OMIM and Orphanet human phenotype–gene associations were obtained from the Jackson Laboratory HPO database (hpo.jax.org, October 2021 release), providing curated links between HPO terms and human genes^28^. The OMIM and Orphanet-based HPO-term gene associations were retrieved for the human abnormal morphology of the great vessels, heart, and CNS phenotypes. Gene-birth-defect associations were also obtained from ClinVar human genetic variants-phenotype submission summary dataset (v2021-11-03)^46^. This dataset was utilized to extract relationships between human genes harboring a pathogenic variant and their associated phenotypes given the birth-defect phenotypes described above. Only genes with pathogenic variants and variants affecting a single gene were considered: That is, variants affecting multiple genes were excluded, due to the complexities in interpreting the relationships between their affected subset of genes and associated human diseases. The ClinVar-based HPO-gene associations were compiled for the human abnormal morphology of the great vessels, heart, and CNS phenotypes. Literature-based human disease–gene associations were obtained from the DISEASES portal^47^. This dataset contains disease–gene associations text-mined from literature and genome-wide association studies. The disease ontology identifier (DOID) and ICD-10 codes listed in this database were converted to HPO terms using mappings directly taken from the Monarch initiative’s Mondo disease ontology^51^. DECIPHER^48^ provided this study with a curated list of genes reported to be associated with developmental disorders, processed by expert clinicians as a part of the Deciphering Developmental Disorders (DDD) study^52^ to facilitate clinical feedback of likely causal variants. The DECIPHER-based HPO-gene associations were compiled for the human abnormal morphology of the heart, and CNS phenotypes. We included a dataset of human congenital heart disease-associated genes associated with syndromic, non-syndromic, and ciliopathic cardiac disorders that was published by the AHA as general guidance for genetic testing by practitioners in 2018^49^. Finally, using the Geneshot API^50^, we queried each one of the 36 CDC birth-defect terms through PubMed to extract PMIDs associated with each term. These PMIDs were then converted into genes using the AutoRIF option of the Geneshot application. The top 50 most frequently occurring genes were retained as gene sets for each birth defect. A file containing all gene-birth-defect associations in JSON format can be retrieved from the ReproTox-KG download page.

### Linking small molecule and drugs to their known targets

Drugs and small molecules that have known targets were extracted from the TCRD database^26^ and converted into KG assertions. Only compounds with a defined structure were included because other substances do not have PubChem^53^ chemical IDs. In addition, only human targets were included, and only single gene/protein targets were included excluding some multi-component ion channels and transporters. Properties such as SMILES, binding affinity, original source, PubChem IDs, and common names are provided for each drug. These drug-target associations are available in JSON format from the ReproTox-KG download page.

### Linking small molecules to genes based on changes in gene expression

The ReproTox-KG holds knowledge about most FDA-approved drugs and over 30,000 preclinical small molecules profiled by the LINCS program for their effects on the transcriptome of selected human cell lines^27^. To extract a set of genes that are up- or downregulated by each drug and small molecule profiled by the L1000 assay for LINCS, we computed the mean of the Characteristic Direction^54^ gene expression vector for each drug in the LINCS L1000 chemical perturbation signature dataset downloaded from SigCom LINCS^55^. We then retained the top 25 up- and downregulated genes for each drug. This allows us to take the top genes affected by the small molecule perturbation without overwhelming the database with differential gene expression associations. All drug–gene associations can be downloaded from the ReproTox-KG download page.

### Drug–drug similarity based on gene expression and chemical structure

To enable drug–drug similarity search across the ReproTox-KG, and to perform the semi-supervised machine learning predictions, we developed two drug–drug similarity matrices, one based on structure and one based on gene expression similarity. The drug–drug similarity matrix based on gene expression vector similarity was computed by transforming the consensus signatures described above using cosine similarity, comparing all pairs of consensus drug gene expression vectors to produce a square matrix where the value at *(i,j)* is the gene expression-based cosine similarity between the drugs at row *i* and column *j*. The matrices that contain the consensus signatures for all drugs and small molecules, and the drug–drug similarity matrix are available for download from SigCom LINCS^55^ and the ReproTox-KG download page. To create drug–drug similarity based on chemical structure similarity, we first converted the SMILES strings of each compound to a binary feature vector using the Morgan fingerprint (2048 bits) method^56^ with radius implemented in RDKit^57^. We also used RDKit Chem module’s QED and Crippen functions for physiochemical properties. Other chemical structure similarity methods such as MACCS, Avalon, Atom Pair, RDKit with maxPath 2 and 4, and Topological fingerprints using FingerprintMol were tested, confirming that the method chosen is justified as superior or comparable. Next, we computed the inverse document frequency (IDF) between all pairs of drug vectors as the distance measure between each pair of drugs. The resultant matrix of drug–drug similarity based on chemical structure is available from the ReproTox-KG download page. Chemical structure-based similarity search was also implemented using a workflow which queries the KG for compounds and generates fingerprints and similarity measures at runtime, for additional flexibility and interoperability.

### Computing frequency of genetic variants

Gene intolerance scores were introduced to the knowledge graph from three main sources: haploinsufficiency, triplo-sensitivity, and general intolerance. Probability of being loss-of-function-intolerant (pLI) scores^58^ for 18,225 human genes were obtained from a large-scale study conducted by the Exome Aggregation Consortium (ExAC)^59^. Residual Variant Intolerance Score (RVIS)^60^ values of 16,956 human genes were adopted from a large-scale analysis that processed 6503 human whole exome sequences made available by the NHLBI Exome Sequencing Project (ESP)^61^. The resulting scores were then compared with information on whether the gene causes any known Mendelian diseases. In this sense, genes with higher functional mutation to total variant site ratios are considered more tolerant^60^. Dosage sensitivity scores^62^ such as haploinsufficiency and triplosensitivity for 17,263 human genes were presented by meta-analyzing 753,994 individuals with neurological disease phenotypes^62^. The provided scores are utilized in gene prioritization by their potential loss-of-function or gain-of-function through the introduction of de novo rare copy-number variants (rCNVs) as opposed to the point mutations.

### Gene–gene similarity based on co-expression

Gene–gene similarity associations were obtained from the human gene–gene correlation matrix provided by the ARCHS4 resource^63^. The matrix stores the Pearson correlation coefficient between genes across bulk RNA-seq expression samples uniformly processed by the ARCHS4 pipeline. Genes were filtered to include only protein-coding genes to keep the size of the graph manageable, and for each of the 17,966 genes, the top five most positively and most negatively correlated genes based on the correlation coefficients were extracted for a total of 170,819 edges. Each edge was weighted by the correlation coefficient between the two connected genes. These gene–gene associations were then integrated into the ReproTox-KG. From these associations, it may be possible to identify genes that are potentially affected by known teratogens and discover the role that different groups of genes may play in inducing birth-defect phenotypes.

### Placental crossing and D and X category predictions for small molecules

Using a semi-supervised learning approach, we generated placental crossing scores and D and X category scores for all FDA-approved and preclinical compounds profiled by LINCS that are included in the ReproTox-KG. To obtain true positives for placental crossing, we first extracted the list of 248 compounds assembled by Di Filippo et al.^64^. Category D and X drugs were obtained from DrugCentral^33^ and Drugs.com^34^, and drugs were filtered by those which could be mapped to the LINCS L1000 compounds through the LINCS small molecule metadata, including drug names and synonyms. Out of the 248 placental crossing drugs, we were able to manually map 143 to the L1000 profiled compounds. Drugs associated with both categories were considered category X.

Predictions were made with the two drug–drug similarity matrices assembled based on gene–gene co-expression correlations, and chemical structure similarity as described above. Predictions were made using the same approach described in a publication describing DrugShot^41^. Specifically, we computed the average distance of each drug to the drugs labeled as positives, and then rank the drugs by this average distance. Importantly, the diagonal is removed to prevent contribution to the average distance from the drug to itself. This approach was used to score all LINCS compounds. The placental crossing scores and the category D and X scores for all drugs and small molecules in the ReproTox-KG are displayed as node properties for drugs and are depicted as the hue level of the drug nodes in the ReproTox-KG user interface. In addition, the predictions are provided as a supplementary table (Supplemental Data 6). To assess the reliability of the similarity scores, a Kolmogorov–Smirnoff-like random walk statistic was applied. Half of the drugs were held-out when constructing the prediction scores for the compounds, and the other half of held-out set of drugs was used to construct a bridge plot and compute a normalized enrichment score (NES). The NES is the enrichment score (ES) divided by the average ES of 10,000 label permutations of those scores.

Two methods were developed to combine the predictions made by the gene expression and chemical structure similarity predictions. Given two scoring vectors produced by the two different similarity matrices, the Top Rank method takes the highest ranking of the drugs across all predictions to be the aggregated score. This score is then used for the bridge plot and normalized enrichment score. Alternatively, given two similarity score vectors, one based on expression and one based on structure, we aggregated these predictions by assigning a weight to each score coming from the two sources: expression and structure. These weights were optimized on a pre-task involving logistic classification of drugs into different mechanisms of action (MOA) using the same gene expression and chemical structure similarity prediction approach. More specifically, a dot product was performed between the two features and a weight matrix, the result is passed through a sigmoid function and the binary cross entropy loss is measured between the output and the true class where 1 means that the drug has the MOA, and 0 means that the drug does not. This is optimized for performance using the Adam optimizer^65^ on 60 different MOAs, for which there are at least 10 drugs, with 10 repeats shuffled randomly; class gradient contributions are weighted to counter the inherent class imbalance. The learned weights are then applied for combining the L1000 and structural features in the FDA drug categorization and placenta crossing sets into a singular score. This score is then used for the bridge plots and the normalized Enrichment Scores. This analysis is performed independently for predicting FDA drug categorization, and for the placenta crossing predictions.

### UMAP visualization of L1000 perturbations

Uniform Manifold Approximation and Projection (UMAP)^66^ was applied to the normalized L1000 count matrix of over 718,055 chemical perturbations performed with different drugs across different cell lines, time points, and concentrations. Perturbations with FDA drug categories D and X and drugs known to cross the placenta were colored by category. To identify the top MOAs in the L1000 perturbation space, we first clustered L1000 perturbations directly using Hierarchical Density-based Spatial Clustering of Applications with Noise (HDBSCAN)^67^ with a minimum cluster size of 40 which struck a qualitative balance between the number of clusters and concordance with apparent clusters of FDA category D and X drugs in the UMAP. We then selected the top 25 clusters with highest concentration of drugs for each drug category, finally we identified the top 5 MOAs for drugs in those clusters. We colored the L1000 UMAP with those top MOAs.

### ReproTox-KG backend KG database

**Fig. 1:**
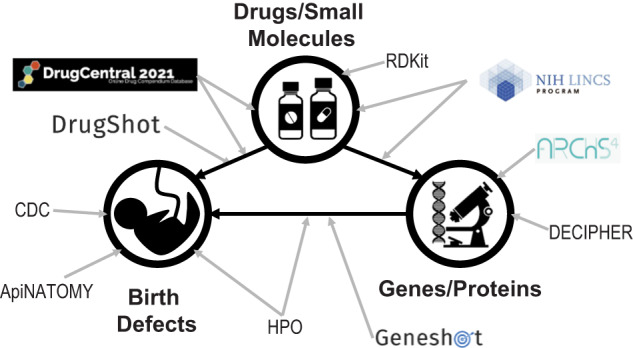
Overview of the ReproTox-KG sources and connections. The ReproTox-KG is made of lists of birth defects extracted from HPO and the CDC and birth-defect gene associations from HPO and Geneshot; HPO is a resource that provides an ontology of human phenotype and the human genes that have evidence to be associated with such phenotype; The CDC website has a dedicated site for listing major birth-defect terms. Using Geneshot birth-defect terms were connected to genes based on co-mentions in abstracts. The ReproTox-KG also has drug/birth-defect associations from DrugCentral, DrugShot, and other sources; To associate birth-defect terms with drugs, DrugShot was used to query birth-defect terms and drug-birth-defect association were determined based on co-mentions in abstracts. In addition, drug–gene associations were taken from the LINCS L1000 data and from drug-target knowledge. The LINCS L1000 data contain drug perturbation followed by expression for ~30,000 drugs and small molecules applied to ten human core cell lines at different concentrations and where gene expression was measured at different time points. Gene–gene associations are based on co-expression from ARCHS4; ARCHS4 contains uniformly aligned RNA-seq data from GEO and the gene–gene co-expression correlations were computed by randomly selecting thousands of RNA-seq sample and computing correlation with the Pearson’s correlation coefficient formula. Drug–drug associations within the knowledge graph are based on structural chemical similarity using RDKit, a software library that contains functions to compute the similarity between compounds based on different representations and algorithms.

The ReproTox-KG uses a graph-structured data model to integrate data. The KG is implemented using Neo4J^21^. The information in the ReproTox-KG represents a network of nodes representing birth defects, genes, and drugs, and edges representing their relationships. In addition, attributes/properties of the nodes and edges are provided. The ReproTox-KG is made up of datasets from the various sources listed above and listed in two tables (Tables 1 and 2) and illustrated in the associated schematic (Fig. 1). The ReproTox-KG uses a standardized JSON schema serialization to ingest data into the KG. Queries to the Neo4J platform are constructed using the Cypher query language^68^.

**Table 1:** ReproTox-KG node and edge sources and enumeration.

Assertion edge type	Name	Relationship	Reference	Nodes/nodes/edges
Birth defect–gene	HPO	Genes known to be associated with a birth defect	Human Phenotype Ontology	599/5093/127,023
	Geneshot	Genes co-mentioned with birth-defect terms in publications	Geneshot	32/1091/1565
Birth defect–drug	FDA Adverse Event Reporting System (Female/Male)	Drugs with reported adverse events related to birth defects	FDA Adverse Event Reporting System	32/94/372
	Drugshot	Drugs co-mentioned with birth-defect terms in publications	Drugshot	679/2207/13,000
Drug–gene	IDG (Drug Target)	Drugs with known human gene targets	Target Central Resource Database	1363/1034/7326
	SigCom LINCS Drug-to-Gene (upregulates/ downregulates)	Drugs that up- or down-regulate genes across the LINCS L1000 signatures	SigCom LINCS	4523/4419/225,509
Drug–drug	LINCS Drugs Cosine Similarity	Drugs that induce similar gene expression patterns across LINCS L1000 signatures based on cosine similarity	SigCom LINCS	4523/3449/20,785
Gene–gene	ARCHS4 (positively/ negatively correlated)	Genes positively or negatively correlated across ARCHS4 gene expression samples	ARCHS4	17,964/12,185/ 170,801

The ReproTox-KG is made of entities (nodes) representing birth defects, genes, and drugs that are connected based on semantic assertions (edges/relationships) extracted from different sources. The table lists the type of assertion, the nature of the relationship, the original source from where the assertion was extracted from, and the number of entities and relations for each entry in the table.

**Table 2:** Attributes of node types and their sources.

Entity type	Property	Source	Description	Entities with property
Birth defect	MedDRA code		MedDRA ontology identifier	32
Drug	Placenta crossing likelihood score	Drugshot	Cosine similarity score to L1000 gene expression signatures for drugs known to cross the placental barrier	4523
	Placenta crossing likelihood rank	Drugshot	Rank (1=most similar) of cosine similarity score to L1000 gene expression signatures for drugs known to cross the placental barrier	4523
	SMILES	PubChem	SMILES structure	4084
Gene	pLI	Exome Aggregation Consortium (ExAC)	Probability of loss-of-function intolerance	9466
	pHI	Collins et al.^62^	Haploinsufficiency score	9466
	pTS	Collins et al.^62^	Triplosensitivity score	9466
	Residual Variant Intolerance Score	NHLBI Exome Sequencing Project	Ratio of functional mutations to total variant sites	9466
	Residual Variant Intolerance Score Percentile	NHLBI Exome Sequencing Project	Percentile of RVIS across all scored genes	9466

The ReproTox-KG is made of entities (nodes) representing birth defects, genes, and drugs that are decorated with attributes and properties associated with them, for example, common identifiers. The table lists the properties and their sources for each entity type, namely, birth defects, genes, and drugs represented in the ReproTox-KG.

### Original graphical user interface to interact with the ReproTox-KG

Since Neo4j currently does not provide an open-source, free, and customizable standalone web-based user interface (UI) to visualize the results from Cypher queries, we developed an original UI with these features for this project. Leveraging the Cytoscape.JS library^69^, the UI renders Cypher query results in JSON format for network visualization. The UI provides the ability to perform queries for finding neighbors of single entities, finding shortest paths between pairs of entities, displaying the networks using various layouts, expanding, and shrinking the size of the displayed subnetwork, viewing properties of nodes and links, and downloading the displayed associations in tabular format.

### Reporting summary

Further information on research design is available in the Nature Portfolio Reporting Summary linked to this article.

## Results

### Overall construction and composition of the ReproTox-KG

**Fig. 2:**
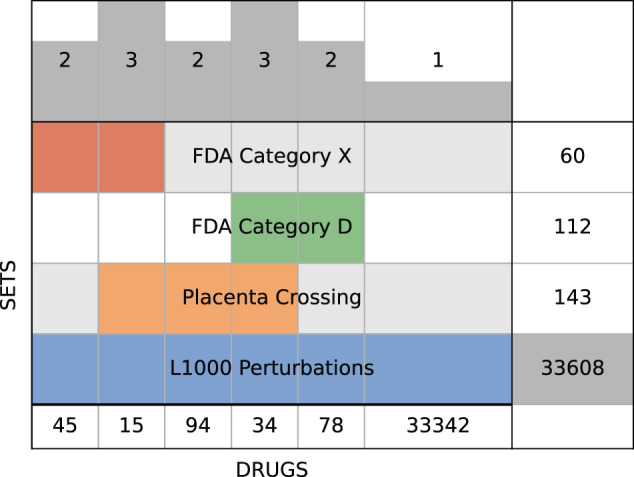
Overlap of drugs across categories. Supervenn diagram of drug identifier overlap between FDA categories D and X, known placenta crossing drugs, and unique drugs and small molecules within the L1000 LINCS perturbation datasets. Drugs and compounds not represented in the L1000 perturbations are not included in the counts.

**Fig. 3:**
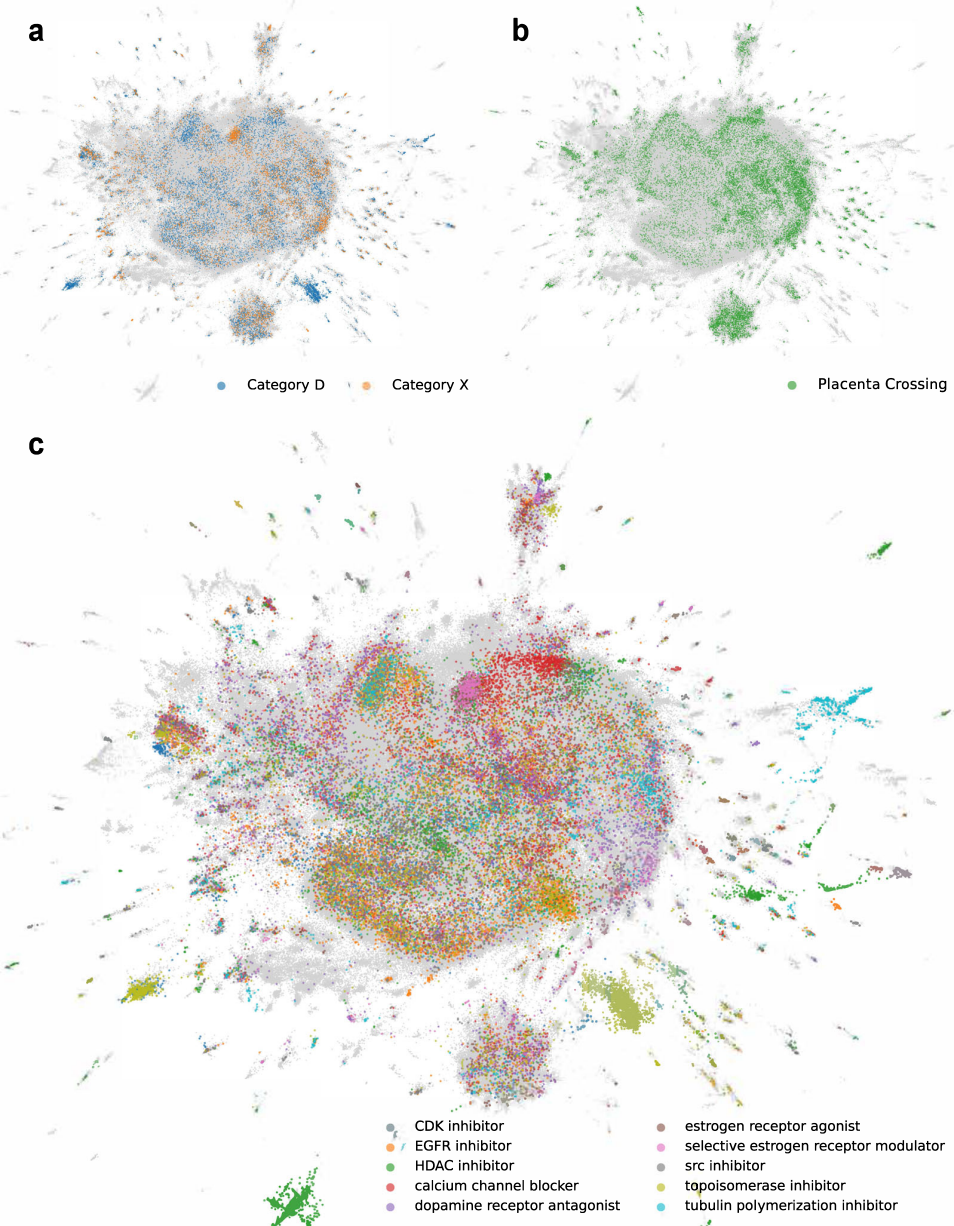
Global visualization of gene expression signature similarity for LINCS drugs. UMAP of 718,055 L1000 perturbations, colored by **a** FDA D and X category; **b** known placental crossing; **c** top MOAs across clusters. Clusters computed using HDBSCAN with a minimum cluster size of 40, top 25 clusters for each category, and top five MOAs of those clusters are included.

The ReproTox-KG contains semantic assertions that connect birth defects, genes, and drugs. In addition, drug–drug and gene–gene similarity assertions are included (Fig. 1 and Tables 1 and 2). Each entity in the ReproTox-KG has a set of attributes and properties. Some of these attributes are unique to the project. For example, we rank the likelihood of all included compounds and drugs to cross the placental barrier and to cause birth defects using a semi-supervised machine learning approach based on similarity matrices that were previously produced and some labeled data. In semi-supervised learning (SSL) most of the data is unlabeled, but some of the data is labeled. The subset of labeled data consists of a list of 248 drugs that are known to cross the placenta^64^, and lists of FDA-approved drugs classified in the X (*n* = 60) and D categories (*n* = 112). We then manually mapped these drug names to small molecules profiled by the LINCS L1000 assay using terms, synonyms, and IDs derived from PubChem^53^ resulting in 143 mapped placenta crossing drugs, as well as 60 X and 112 D category drugs (Fig. 2). Next, we constructed two drug–drug similarity matrices, one based on drug structural similarity, and one based on gene expression induced signature similarity. These matrices are used to perform semi-supervised machine learning to prioritize all drugs for the likelihood to cross the placenta or to be categorized as D and/or X. Before performing such predictions with these two matrices, we projected the category D and X drugs (Fig. 3a) and the known placental crossing drugs (Fig. 3b) onto the LINCS L1000 gene expression space of 718,055 gene expression signatures induced by >30,000 small molecules using UMAP^66^. We observe that these drugs fall into distinct regions within the L1000 gene expression space. By comparing the UMAP visualization of the known placental crossing drugs and the category D and X drugs to the same layout with highlighted known mechanisms of actions (Fig. 3c), we observe that dense clusters of D and X drugs involve estrogen disruptors and topoisomerase inhibitors. Other clusters colored by their unique MOAs also have many placental crossing drugs and the category D and X drugs within them. The observed punctate distribution strongly suggests that we can make predictions about the likelihood of preclinical drugs to induce birth defects and cross the placenta. The clinical relevance of these predictions needs to be qualified by considering additional factors, such as the impact of influx (solute carrier) proteins, efflux (e.g., ABC) transporters, as well as in situ metabolism mediated by, e.g., cytochrome P450 enzymes.

**Fig. 4:**
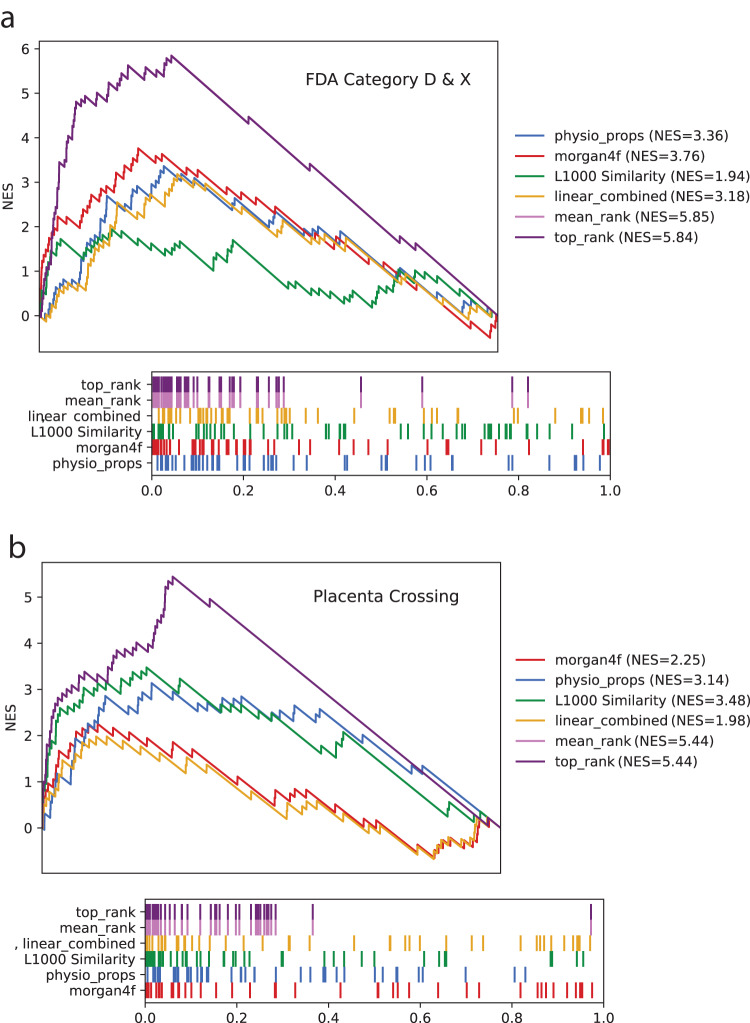
Drug category and placental crossing prediction performance. Bridge plots colored by prediction method for (**a**) predicting FDA D and X categories; and (**b**) placenta crossing. The NES are shown in the legend. Leading edges of the same bridge plots are shown on the right of each complete bridge plot.

Next, we apply the semi-supervised learning approach to rank all mapped approved drugs and preclinical compounds to estimate their likelihood to cross the placental barrier, and to induce a birth defect. We use the category D or X drugs and the drugs known to cross the placenta as the true positives. The predictions are based on how close the other drugs are to these drugs in feature space. Such feature space is defined based on chemical similarity, gene expression similarity, or a combination of them. During benchmarking, we hold out a third of the drugs for testing. We observe that with the L1000 signature similarity matrix alone we achieve an NES of 1.94 for predicting D and X category membership and 3.48 for placental crossing (Fig. 4). The predictions that are based on chemical structural similarity alone achieve NES of 3.76 and 3.14 for D and X category membership and for placental crossing, respectively (Fig. 4). Combining the predictions made by the gene expression data together with the chemical structure data with the Top Rank or Mean Rank methods improves such predictions to 5.85 for D and X category membership, and 5.44 for the placental crossing predictions. Overall, these are high-quality predictions for a semi-supervised approach. Importantly, these predictions perform well at the leading edge (Fig. 4a, b). It should be noted that predictions made with structural similarity only perform well when we define the similarity between compounds using IDF instead of Tanimoto. This is likely because there is a bias with the Tanimoto method which emphasizes similarity between complex larger compounds that share common features. The predictions made by the semi-supervised method highly rank compounds that are known as ACE inhibitors, antibiotics, and statins (Tables 3 and 4). This is not surprising because such compounds are already common among known category X & D drugs^70^ and drugs that are known to cross the placenta. For example, the top ranked drug by structural similarity to be categorized as X and D is enalaprilat. Enalaprilat, an ACE inhibitor, is the active metabolite of the oral drug, enalapril. Both are used to treat high blood pressure^71^. They are listed as category C for the first trimester and as category D for the second and third. ACE inhibitors also cause fetal renal failure resulting in oligohydramnios. These birth defects are deformations caused by uterine pressure on the fetus in the second and third trimesters. Overall, such predictions can be used to warn about the potential of newly approved drugs to cross the placenta and induce birth defects, but it should be noted that these predictions cannot be taken as a substitute for experimental confirmation.

**Table 3:** Top predicted X and D category drugs and preclinical compounds.

Drug	D & X L1000	D & X structure	Weighted	Top rank	D known	X known
Enalaprilat		1	1	1	0	0
TAK-715	1			1	0	0
Pitavastatin		2	11	2	0	0
BRD-K08703257	2			2	0	0
Ramipril		3	2	3	1	0
Pentoxifylline	3			3	0	0
Trandolapril		3	6	4	1	0
Troglitazone	4			4	0	0
Perindopril		4	3	5	1	0
FTI-276	5			5	0	0
BRD-K76846644		5	5	6	0	0
Gossypetin	7			6	0	0
Pravastatin		6	4	7	0	1
Phenothiazine	9			7	0	0
Evodiamine		7	7	8	0	0
Lorazepam	10			8	1	0
BRD-K40167599		8		9	0	0
Losartan	11			9	1	0
Lovastatin		9		10	0	1
Zebularine	13			10	0	0
CTPB		10	15	11	0	0
Risperidone				11	0	0
VX-765		11	8	12	0	0
BMS-191011				12	0	0
Methiopril		12	10	13	0	0
EX-527				13	0	0
Canrenone		13	12	14	0	0
BRD-K55591206				14	0	0
BRD-K80672993				15	0	0
Oxytetracycline		14		15	0	0
BRD-K63784565			9		0	0
Zofenopril-calcium			13		0	0
BRD-K93367411			14		0	0
BRD-K08533345	14				0	0
BRD-K27194553	12				0	0
BRD-K27889081	8				0	0
BRD-K37848780	15				0	0
BRD-K76551930	6				0	0
Imidapril		15			0	0

The top-15 ranked compounds predicted using semi-supervised learning with L1000 gene expression similarity or chemical structure similarity, or two by two methods that combine the predictions from the two sources, namely, weighted, and top rank, are listed together with whether these were previously known to belong to the X or D categories.

**Table 4:** Top predicted placental crossing drugs and preclinical compounds.

Drug	L1000	Structure	Weighted	Top rank	Known
Nafcillin		1	1	1	0
FTI-276	1			1	0
Piperacillin		2	4	2	0
Gossypetin	2			2	0
Cefotaxime		3	3	3	0
TAK-715	3			3	0
Ciclacillin		4	2	4	0
BRD-K08703257	4			4	0
7-aminocephalosporanic-acid		5	6	5	0
Temozolomide	5			5	0
Penicillin		6	5	6	0
CGS-21680	6			6	0
Ceforanide		7	9	7	0
Y-27632	7			7	0
Lorazepam	8			8	1
BRD-K43966364		8	8	9	0
Benzathine		8		9	0
Estradiol-cypionate	9			10	0
BRD-K50776152	10			11	0
Isoetharine		9		11	0
Enalaprilat		10	12	12	0
Rolipram	11			12	0
BRD-A66025870		11		13	0
PT-630	12			13	0
EMF-csc-9	13			14	0
Practolol		12		14	0
Cephalothin		13	15	15	1
DL-TBOA	14			15	0
Pravastatin		14	7		0
Cefoperazone			10		1
Orciprenaline			11		0
Ampicillin			13		1
Dicloxacillin			14		0
BRD-K55591206	15				0
Micropenin		15			0

The top-15 ranked compounds predicted using semi-supervised learning with L1000 gene expression similarity or chemical structure similarity, or two by two methods that combine the predictions from the two sources, namely, weighted, and top rank, are listed together with whether these were previously known to cross the placenta.

In addition, for each gene included in the KG, we computed likelihood for deleterious mutations using three established methods: (pLI) scores^58^, RVIS^60^, and dosage sensitivity scores [20]. Each entity in the ReproTox-KG also includes links out to databases based on entity ID resolution. In particular, 694 birth defects are mapped to HPO identifiers^28^, 18,233 genes and proteins are mapped to HGNC IDs, and 5403 drugs are mapped to their PubChem identifiers^53^. Lists of birth-defect terms were extracted from HPO^28^ and the CDC website^29^. The 127,023 associations between birth-defect terms and genes were extracted from OMIM^44^, Orphanet^45^, ClinVar^46^, DISEASES^33^, DECIPHER^48^, American Heart Association (AHA)^49^ and Geneshot^50^. The 13,561 assertions between birth defects and drugs were extracted from DrugCentral^50^, and DrugShot^41^. Two types of assertions connect genes and drugs within the ReproTox-KG: 1) genes that are differentially expressed after drug treatment based on transcriptomics, and 2) known drug targets for the drugs. Overall, 225,509 drug–gene associations were extracted from the LINCS L1000 data^55^, and 7326 drug-target assertions were extracted from Pharos^26^. Similarly, 9546 drug–drug similarity assertions are identified based on chemical similarity and 33,608 based on gene expression signature similarity. Finally, gene–gene similarity included in the KG is based on gene–gene co-expression^63^.

**Fig. 5:**
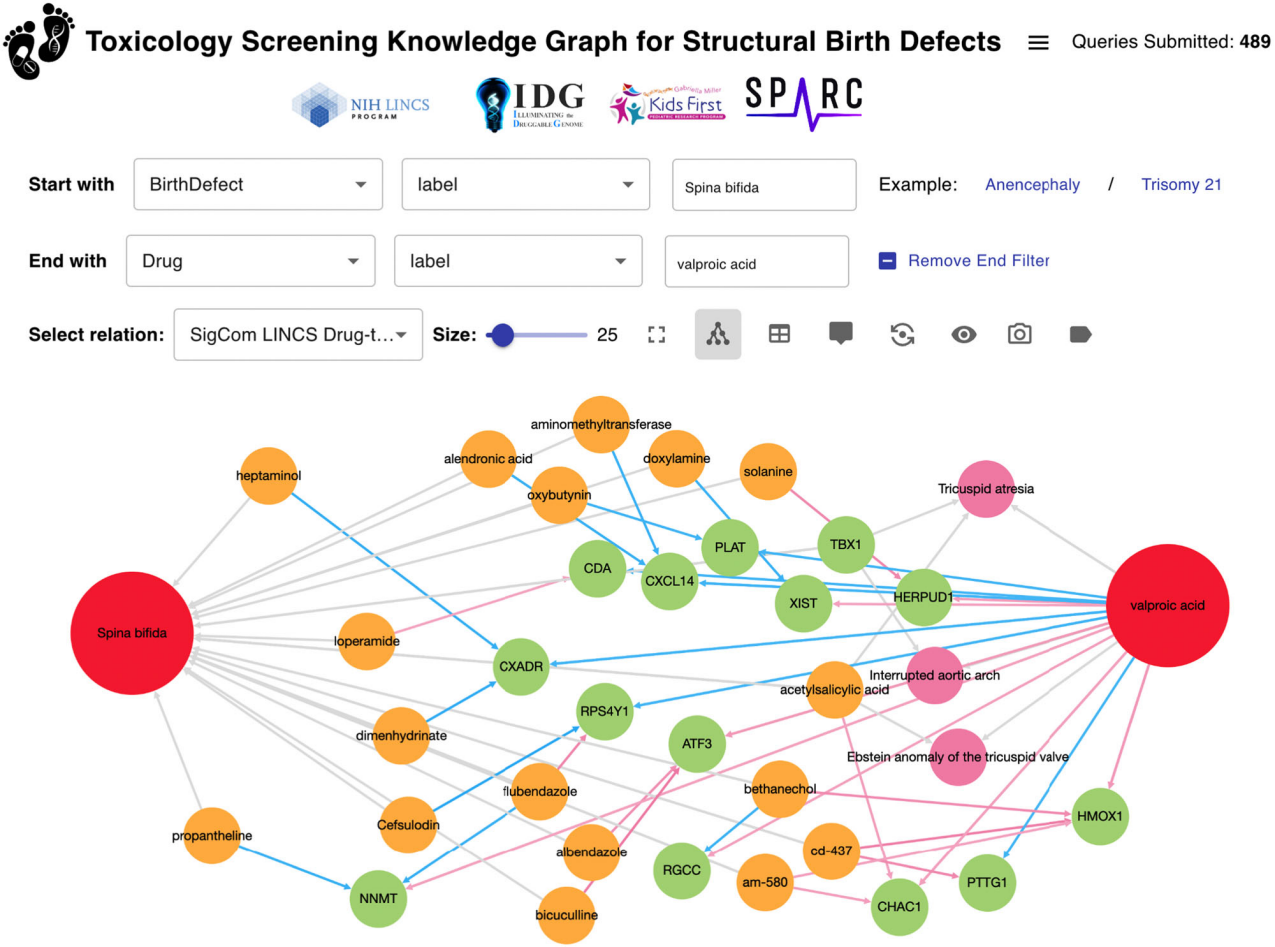
Screenshot from the ReproTox-KG user interface. A query to identify connections between the birth defect Spina Bifida and the drug valproic acid with a limit of 25 nodes is provided as an example.

The processed data from these resources was created by customized extract, transform, and load (ETL) scripts and stored as a JSON schema data model. This processed data was ingested into a Neo4J database, and it is made available for download on the ReproTox-KG website at: https://maayanlab.cloud/reprotox-kg/downloads. The ETL scripts are open-source and available from: https://github.com/nih-cfde/ReproToxTables/. To provide access to the processed data in a user-friendly manner, we developed an original graphical user interface (Fig. 5). The example shown provides possible links that connect valproic acid and Spina Bifida, a known association^72^. The interface can be accessed from https://maayanlab.cloud/reprotox-kg.

### Extraction of birth-defect gene-small molecule cliques to explain potential MOAs

**Fig. 6:**
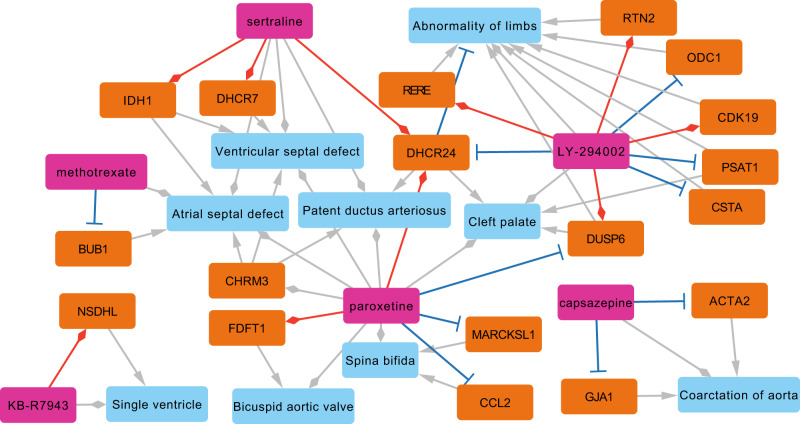
Network of cliques connecting drugs, birth defects and genes. Cliques are made of drugs that have a placenta crossing predicted rank of less than 3000 and are known to induce a birth defect based on literature evidence. These drugs are connected to the genes that their expression is affected by the drugs based on LINCS L1000 data. Finally, associations between genes and birth defects are established based on known mutations that are known to cause the same birth defect. Light blue nodes represent birth-defect terms, orange nodes represent genes, and pink nodes represent drugs and preclinical small molecules. Red lines with diamond-heads indicate an L1000 consensus drug signatures that upregulates the gene, and plungers indicate an L1000 consensus drug signature that downregulates the target gene. Gray arrowheads indicate genes that their mutations induce a birth defect, and gray diamond-heads connect drugs to the birth defects they are known to induce.

To demonstrate the utility of the ReproTox-KG to illuminate knowledge, we queried the graph to identify all three-node cliques. That is, we extracted from the ReproTox-KG all instances where a birth defect was connected to a gene and a drug that are also connected. In total, 533 such cliques are identified (Supplemental Data 7). From this collection of cliques, there are cliques for six drugs and small molecules that were not previously listed as crossing the placenta and have a placental scoring rank of less than 3000 (out of 30,000) (Fig. 6). This subnetwork demonstrates how the ReproTox-KG can be used to suggest MOA for how these drugs may induce specific birth defects by affecting the gene expression of genes known to be associated with the birth defect. For example, LINCS L1000 transcriptomics data show that the approved drug methotrexate, a chemotherapeutic and immunosuppressive drug, inhibits the expression of the mitotic checkpoint serine/threonine-protein kinase BUB1. *BUB1* is known to cause microcephaly when mutated^73^, and methotrexate is known to cause microcephaly and atrial defects^74^. Hence, this adverse effect of methotrexate can be attributed to its direct influence on the expression levels of *BUB1*. It should be noted that BUB1 is a critical component of the cell cycle pathway^75^. Hence, it is likely that methotrexate interferes with a specific stage of development that requires cell proliferation via indirect downregulation of *BUB1*. Similarly, the experimental drug LY-294002 which is a morpholine-containing chemical compound that is a strong inhibitor of PI3K, was previously shown to influence cell proliferation of epithelial cells isolated from human fetal palatal shelves (hFPECs)^76^. Besides inhibiting the activity of PI3K, LY-294002 increases the expression of *DUSP6*, a dual specificity phosphatase that dephosphorylates members of the PI3K pathway. There is evidence that PI3K phosphorylates DUSP6, and this phosphorylation induces DUSP6 degradation^77^. Such observation is consistent with the ReproTox cliques’ subgraph.

The approved antidepressant drug sertraline is reported to induce cardiac and vascular birth defects based on analysis of FAERS^78^. The ReproTox-KG subnetwork of cliques suggests that such adverse birth defects could be mediated via the activation of the dehydrocholesterol reductase *DHCR7* and *DHCR24*^79^. These two enzymes are critical components of the cholesterol biosynthesis pathway. Mutations in *DHCR7* are known to cause Smith–Lemli–Opitz syndrome, a disease of multiple congenital abnormalities^80^, while mutations in *DHCR24* can cause desmosterolosis^81^. Hence, it is plausible that sertraline mediates induction of cardiac and vascular birth defects via up-regulatory effects on *DHCR7* and *DHCR24*. Unlike methotrexate, sertraline is currently not contraindicated in pregnancy and it is classified as a category C drug. Taken together, ReproTox-KG evidence suggests that pregnancy should be listed as a contraindication for sertraline. Overall, these are just a few examples of how the ReproTox-KG can illuminate knowledge about potential mechanisms of how drugs and preclinical small molecules may induce birth defects.

## Discussion

Currently, in the clinical genomic diagnostic research setting, methods for prioritizing variants and genes for association with birth defects are done by utilizing databases such as ClinVar^82^, ClinGen^83^, as well as gene lists related to birth defects from other annotated resources. In contrast, the ReproTox knowledge graph links evidence about associations between chemicals, phenotypes, and genes. This data integration effort offers hypotheses about drugs and compounds that potentially could be involved in the induction of birth defects. There are many cases where there are no causative genetic variants that have been attributed to birth defects. It is possible that exposure to certain teratogens could either alone or via interaction with gene variants potentially cause birth-defect phenotypes. Such interactions may be illuminated by the ReproTox knowledge graph. In addition, the ReproTox knowledge graph has the potential to make an impact on the field of regulatory toxicology by identifying teratogenic potential for preclinical compounds, including their potential MOA to induce such birth defects. Currently, agencies such as the FDA and EPA are faced with the pressures of an increase in applications and a greater demand for making decisions without animal testing.

To characterize associations between small molecule compounds and their potential to induce reproductive toxicity, we gathered knowledge from multiple sources to construct a reproductive toxicity knowledge graph with an initial focus on associations between birth defects, drugs, and genes. The idea of abstracting genes, drugs, and diseases into networks is not new. We and others have pioneered the construction of networks to represent functional and physical associations between genes/proteins^84–86^, drugs and their targets^87,88^, and diseases based on their gene set similarity^89^. The unique feature of the ReproTox-KG is that it provides a flexible framework not only to connect entities such as gene-drug, gene–gene, gene-birth defect, drug–drug, and drug-birth defects, but also to query this network, extend it, visualize it, and add attributes to different node types.

Similar efforts to ReproTx-KG have recently been published, including studies that attempted to use graph embedding Deep Learning algorithms to predict missing associations between drugs and diseases^90^, drug repurposing opportunities^91,92^, predicting drug targets^93,94^, adverse events^95^, and drug–drug interactions^96^. These are just a few studies in this domain. Here, we did not attempt to make such graph-based predictions but provided the needed building blocks to enable such future applications. Hence, the ReproTox-KG was developed as a resource for the community to explore and expand.

One of the limitations of knowledge graphs is their inability to cover many associations between many entities. For example, we decided to only consider the top 25 up and downregulated genes for each drug. This leaves out many genes that may be affected by drugs. These genes will be missed from queries and post-hoc analyses. We also created consensus signatures for each drug from the LINCS L1000 data. Computing such consensus masks the effect of drugs in specific cellular contexts. To make the ReproTox-KG project focused and manageable, we kept its scope limited. However, tissue and cell type distribution of the affected genes, and how drugs and small molecules induce such differential effects, are critical information for associating genes and drugs with birth defects. Such information is partially available and could be included in future releases of ReproTox-KG. The L1000 transcriptomics dataset is not a mainstream assay, but the data produced by this assay provides a glimpse to the effect of many drugs and preclinical small molecules on human cells. Before such data became available, it was mostly unknown whether drugs that are labeled with the same known MOAs, or having the same targets, will induce similar gene expression patterns when applied to human cell lines. Our observation, that in general, we can see that gene expression signature clusters by the same known MOAs is profound. Another excellent resource for gene expression during development is DESCARTES, a human cell atlas for fetal tissues^97^. Such a dataset should be considered in future studies because it provides gene expression across tissues at different stages of development. Understanding the time window of the expression of a gene in a specific tissue during development is critical for better elucidating the molecular mechanisms of many birth defects. It also should be noted that the ReproTox-KG is preliminary and should not be used for clinical applications and clinical decision support.

## Supplementary information

Description of Additional Supplementary Files

Supplemental Material

Supplementary Data 1

Supplementary Data 2

Supplementary Data 3

Supplementary Data 4

Supplementary Data 5

Supplementary Data 6

Supplementary Data 7

Reporting Summary

## Data Availability

All files needed to reconstruct the knowledge graph with instructions are available from https://maayanlab.cloud/reprotox-kg/downloads. All additional processed data files used for the analysis are available from https://maayanlab.cloud/reprotox-kg/downloads. Initial versions of those files, scripts used to process the data, and links to the original sources are available from https://github.com/nih-cfde/ReproToxTables. The interactive web interface to access the knowledge graph is available at https://maayanlab.cloud/reprotox-kg. All the data used in the study is processed from other primary sources. All other data are available from the corresponding author on request.

## Code availability

The scripts used to process the data are available from https://github.com/nih-cfde/ReproToxTables. Code needed to reconstruct the knowledge graph locally is available from https://maayanlab.cloud/reprotox-kg/downloads. Code needed to recreate the figures can be found at https://github.com/MaayanLab/ReproToxPaper. An alternative method to compute chemical similarity: https://github.com/unmtransinfo/CFChemDb. Snapshot of the code and processed datasets are available from 10.5281/zenodo.8049261^98^.
