# Supplementary material for: Toxicology knowledge graph for structural birth defects
Source: Commun Med (Lond). 2023 Jul 17;3:98. doi: 10.1038/s43856-023-00329-2 (PMC10352311; doi:10.1038/s43856-023-00329-2)
Supplement: Supplementary file 1 — Description of Additional Supplementary Files [file 43856_2023_329_MOESM1_ESM.pdf]

Description of Additional Supplementary Files

File Name: Supplementary Data 1

Description: Observed frequencies of birth defect cases from the KidsFirst cohort data. Each birth defect is mapped to a Human Phenotype Ontology identifier.

File Name: Supplementary Data 2

Description: Birth defect phenotype terms for the great vessels, heart, and central nervous system.

File Name: Supplementary Data 3

Description: Major birth defects listed on the Centers for Disease Control and Prevention (CDC) website as of January 6, 2022. Birth defects were manually mapped to Human Phenotype Ontology identifiers

File Name: Supplementary Data 4

Description: Sets of drugs associated with birth defects extracted from various resources and publications.

File Name: Supplementary Data 5

Description: Drug-birth defect relationships extracted from the FDA Adverse Event Reporting System (FAERS).

File Name: Supplementary Data 6

Description: . Category D, Category X, and placental crossing scores for all FDA-approved and preclinical compounds profiled by the LINCS program

File Name: Supplementary Data 7

Description: Birth defect-gene-drug cliques extracted from the ReproTox-KG. Each row represents an instance where a birth defect, drug, and gene are all interconnected
