# Supplementary material for: Toxicology knowledge graph for structural birth defects
Source: Commun Med (Lond). 2023 Jul 17;3:98. doi: 10.1038/s43856-023-00329-2 (PMC10352311; doi:10.1038/s43856-023-00329-2)
Supplement: Supplementary file 2 — Supplemental Material [file 43856_2023_329_MOESM2_ESM.pdf]

Supplementary Information

FOR

Toxicology Knowledge Graph for Structural Birth Defects

John Erol Evangelista[1,+], Daniel J. B. Clarke[1,+], Zhuorui Xie[1], Giacomo B. Marino[1], Vivian Utti[1], Sherry L. Jenkins[1], Taha Mohseni Ahooyi[2], Cristian G. Bologa[3], Jeremy J. Yang[3], Jessica L. Binder[3], Praveen Kumar[3], Christophe G. Lambert[3], Jeffrey S. Grethe[4], Eric Wenger[2], Deanne Taylor[2], Tudor I. Oprea[3], Bernard de Bono[5], Avi Ma'ayan[1,*]

[1]Department of Pharmacological Sciences, Mount Sinai Center for Bioinformatics, Icahn School of Medicine at Mount Sinai, New York, NY 10029 USA

[2]The Children's Hospital of Philadelphia, Department of Biomedical and Health Informatics; Department of Pediatrics, University of Pennsylvania Perelman School of Medicine, Philadelphia, PA 19104, USA

[3]Department of Internal Medicine, Division of Translational Informatics, University of New Mexico, Albuquerque, NM 87131

[4] Department of Medicine, University of California San Diego, La Jolla, CA 92093, USA

[5]Auckland Bioengineering Institute, University of Auckland, Auckland, New Zealand

[+]Contributed equally.

[*]To whom correspondence should be addressed: avi.maayan@mssm.edu

Fig. S1

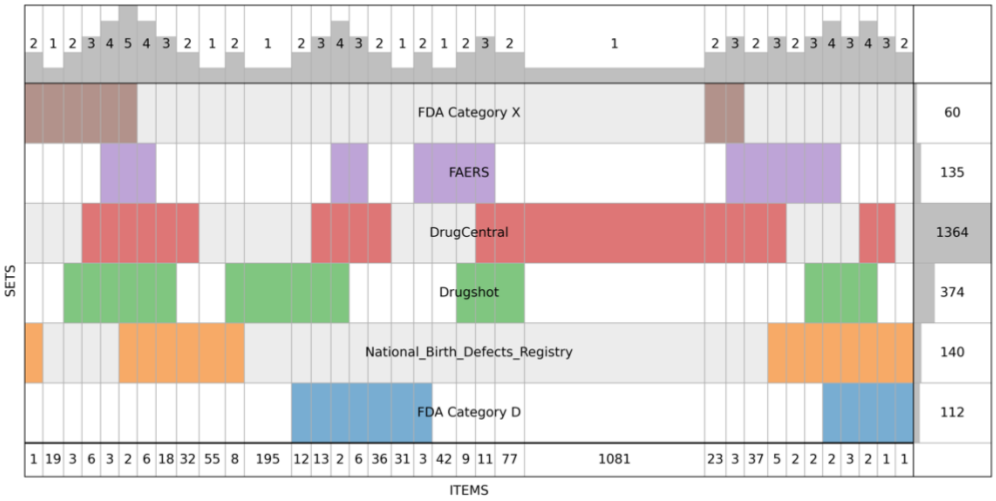

Fig. S1 Visualization of the overlap across sources that report known and potential teratogens. Note that the DrugCentral set is all FDA approved drugs for comparison. The reason Drugshot has low overlap with the DrugCentral list is because it reports all LINCS compounds regardless of whether these are FDA approved.
